# First person – Jing-Fu Bao

**DOI:** 10.1242/dmm.052779

**Published:** 2026-02-03

## Abstract

First Person is a series of interviews with the first authors of a selection of papers published in Disease Models & Mechanisms, helping researchers promote themselves alongside their papers. Jing-Fu Bao is first author on ‘ C-C motif receptor 2 is a core pro-fibrotic factor in uremic cardiomyopathy’, published in DMM. Jing-Fu conducted the research described in this article while a postdoc in the lab of Aiqing Li at Southern Medical University, Guangzhou, China, investigating the pathogenesis of and therapeutic strategies for uremic cardiomyopathy.


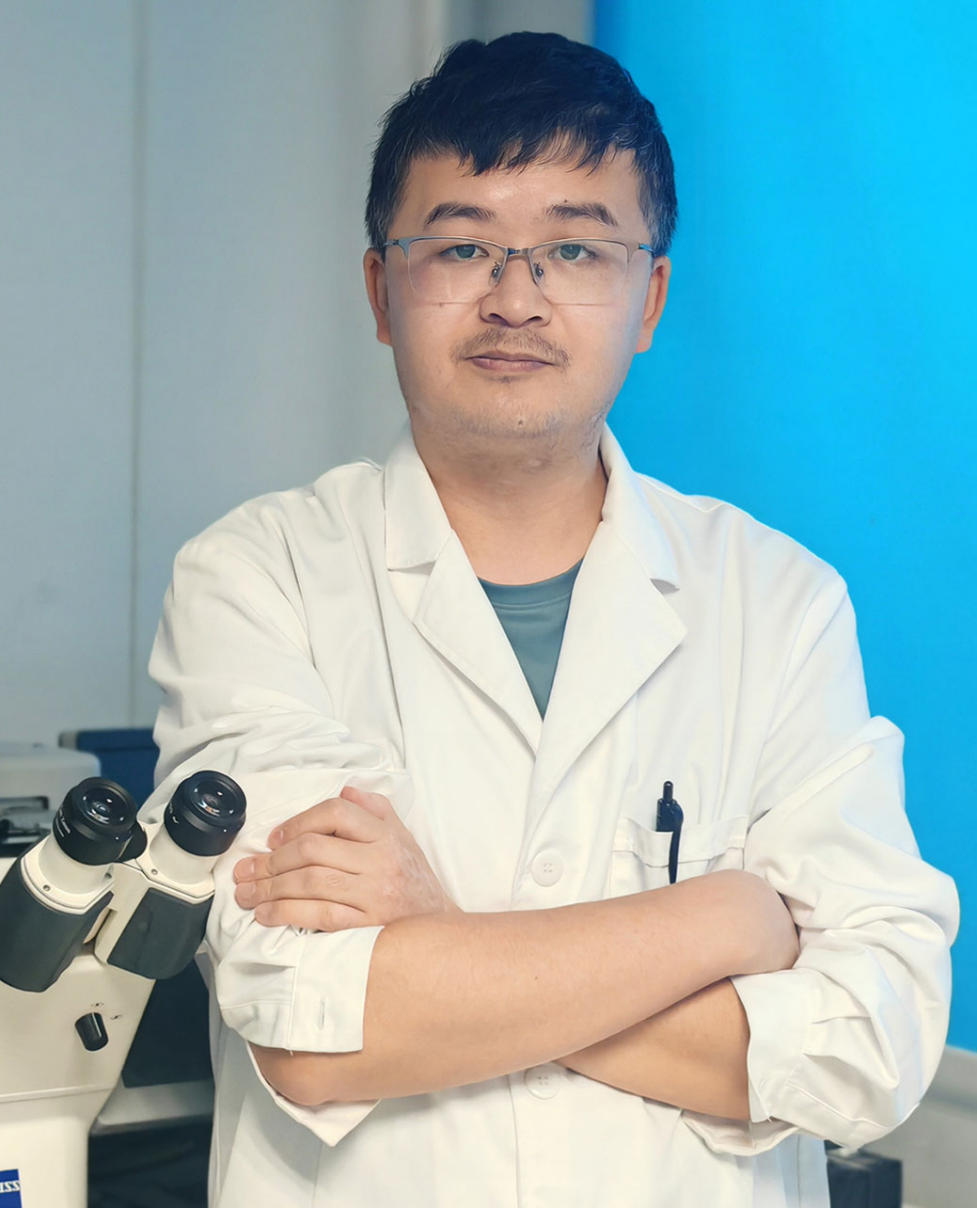


**Jing-Fu Bao**

**Who or what inspired you to become a scientist?**

I spent a lot of time studying physics during my senior high-school period; this sophisticated subject presented a wonderful picture of our world. Since that time, experiencing the beauty of natural law through investigating it seemed like a powerful driver for me to become an academic researcher.

**What is the main question or challenge in disease biology you are addressing in this paper? How did you go about investigating your question or challenge?**

The present study rooted in an unexpected finding in our previous investigation, which revealed that classical 5/6 nephrectomy is unable to simulate human phenotypes of cardiovascular complications caused by chronic kidney disease (CKD) well. Therefore, we aimed to develop better methods for simulating human CKD-induced cardiovascular complications. With an expanding excision range of renal tissues, mice not only demonstrated phenotypes of uremic cardiomyopathy but exhibited hyperphosphatemia without a high-phosphorus diet. In addition, we also improved adenine-induced CKD models by adding a normal diet after a sufficient adenine diet, observing that this overcame malnutrition, which causes smaller heart and inhibits cardiac hypertrophy. For further investigations, we performed RNA sequencing to uncover the cardiac molecular differences between nephrectomy- and adenine-induced CKD, and not only found the differences in the two models but also identified a novel therapeutic target [C-C chemokine receptor 2 (CCR-2)] for uremic cardiomyopathy. Interestingly, inhibiting CCR-2 in an early period of uremic cardiomyopathy caused left ventricular dysfunction despite mitigating cardiac fibrosis, suggesting that an appropriate time point for inhibiting CCR-2 is crucial for its clinical effects.

**How would you explain the main findings of your paper to non-scientific family and friends?**

Nutritional status may be a critical factor affecting the progression of cardiovascular diseases related to CKD. Although sufficient nutrition supply may reduce the mortality risk in patients with CKD, it increases susceptibility to cardiovascular diseases in these patients. Perhaps future studies will identify dietary factors reducing cardiovascular diseases in patients with CKD and provide novel dietary therapies for CKD. Moreover, inflammation is another factor causing cardiovascular diseases in CKD, and mitigating inflammation by inhibiting an inflammation-related core factor – namely, CCR-2 – may represent another therapeutic strategy for cardiovascular complications in patients with CKD.

… cardiac inflammation is important for early response to renal injury …

**What are the potential implications of these results for disease biology and the possible impact on patients?**

We hope that these results lead to concentration on dietary interventions and anti-inflammation in the area of CKD-related cardiovascular complications. Importantly, we propose that cardiac inflammation is important for early response to renal injury, and mitigating inflammation avoids disturbing this indispensable process should it be considered in the treatment of CKD. In addition, some patients with mild renal injury and hypertension demonstrate phenotypes of dilated cardiomyopathy, which may imply a weaker inflammatory response under pathological stimulation.

**Figure:**
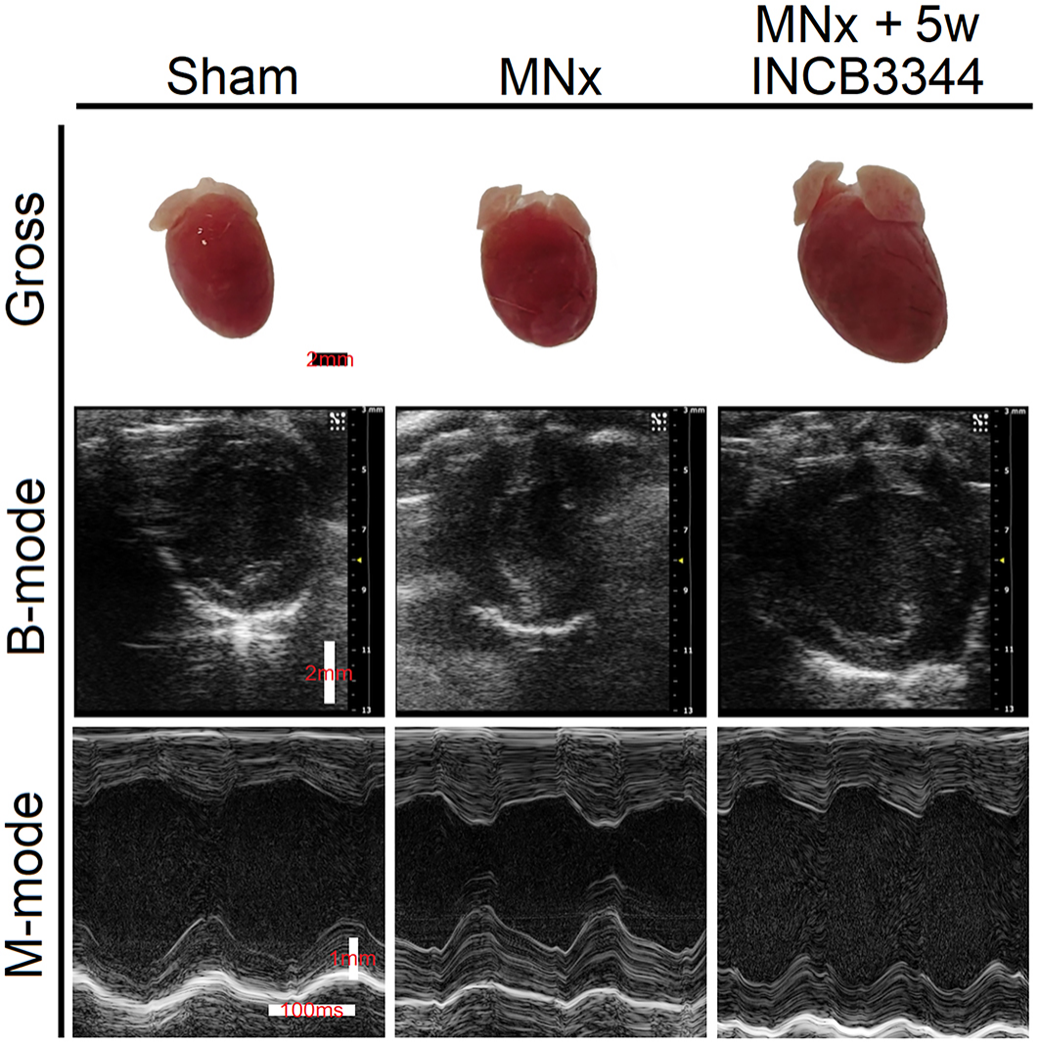
**Prematurely inhibiting C-C chemokine receptor 2 (CCR-2) causes left ventricular dilation and systolic dysfunction in a mouse model of uremic cardiomyopathy.** MNx, modified nephrectomy; INCB3344, small-molecule inhibitor of CCR-2; 5w INCB3344, 5-week INCB3344 injection initiated immediately after nephrectomy surgery.

**Why did you choose DMM for your paper?**

This study combined disease model development and translational research, and we believed it well fits the aim of DMM. Therefore, we felt that DMM will be a good home for our paper as it provides a well-known platform to describe animal models of human disease.

**Given your current role, what challenges do you face and what changes could improve the professional lives of other scientists in this role?**

The main challenge for young independent researchers in China is how to find a suitable unique research direction and obtain their first research funding. Perhaps training aesthetic judgement to find the beauty of natural law during the research process, which aims to improve the ‘taste’ for science, is crucial for identifying the unique pathway for young independent researchers. In addition, excellent funders with a higher scientific appreciation are crucial for the growth of young independent researchers in China.

The main challenge for young independent researchers in China is how to find a suitable unique research direction and obtain their first research funding

**What's next for you?**

Now I have ended my postdoc career, and finding an appropriate position is my next step. I prefer to find a research position in a research-oriented hospital, aiming to perform translational studies on cardiovascular complications caused by CKD. In the future, further investigating how CCR-2 blockade and novel dietary therapies can mitigate cardiovascular disease in CKD is my core mission.

**Tell us something interesting about yourself that wouldn't be on your CV**

Aside from working, I love reading books, especially philosophical books. These books not only provide inspirations for my academic career but help me to find my inner self. Paradoxically, although I really enjoy investigating the biological world through empirical approaches, seeking myself on the basis of mystical paths (compared with empirical approaches) is important to me.
